# Mind wandering “Ahas” versus mindful reasoning: alternative routes to creative solutions

**DOI:** 10.3389/fpsyg.2015.00834

**Published:** 2015-06-17

**Authors:** Claire M. Zedelius, Jonathan W. Schooler

**Affiliations:** Department of Psychological and Brain Sciences, University of California, Santa Barbara, Santa BarbaraCA, USA

**Keywords:** mindfulness, mind wandering, creativity, insight problem solving, analytic problem solving, compound remote associates problems

## Abstract

Based on mixed results linking both mindfulness and its opposing construct mind wandering to enhanced creativity, we predicted that the relationship between mindfulness and creativity might depend on whether creative problems are approached through analytic strategy or through “insight” (i.e., sudden awareness of a solution). Study 1 investigated the relationship between trait mindfulness and compound remote associates problem solving as a function of participants’ self-reported approach to each problem. The results revealed a negative relationship between mindfulness and problem-solving overall. However, more detailed analysis revealed that mindfulness was associated with impaired problem solving when approaching problems with insight, but increased problem solving when using analysis. In Study 2, we manipulated participants’ problem-solving approach through instructions. We again found a negative relationship between mindfulness and creative performance in general, however, more mindful participants again performed better when instructed to approach problems analytically.

## Introduction

The question of what makes some individuals more creative than others has long fascinated researchers and laypeople alike. Interestingly, stereotypes of creative individuals seem to fall into two very different categories. On the one hand, creative individuals are often made out as being creatively successful because they exhibit high levels of concentration and are able to devote their full attention to their creative work. On the other hand, creative individuals are often portrayed as highly volatile and easily distractible characters with scattered minds. These stereotypes describe remarkably different styles of thinking. The first description suggests a tendency toward *mindfulness*, which is often defined as the tendency to attend to and be aware of present moment experiences (Brown and Ryan, 2003; Brown et al., 2007). The second description conveys a tendency toward *mind wandering*, or frequently and often without conscious intention shifting attention away from the present environment (Smallwood and Schooler, 2006; Schooler et al., 2011)^1^. Mindfulness and mind wandering can be viewed as opposing constructs (Mrazek et al., 2012b). In the context of creativity, this opposition raises an intriguing question: Which style of thinking is conducive to being creative? Or is it possible that both mindfulness and mind wandering have distinct benefits for creativity?

## Mind Wandering and Creativity

Previous research provides mostly indirect evidence for a relationship between individual differences in traits related to mindfulness or mind wandering and creativity. For instance, individuals with attention deficit hyperactivity disorder (ADHD), which is known to be associated with increased mind wandering (e.g., Shaw and Giambra, 1993; Barkley, 1997), tend to score higher on creativity tests and report more creative achievements in their everyday life than individuals without ADHD (White and Shah, 2006, 2011). Moreover, individuals with reduced latent inhibition, which is defined as the ability to screen previously irrelevant content from conscious awareness, score higher on creativity tasks such as the generation of novel and useful ideas (Carson et al., 2003; Fink et al., 2012), and lifetime creative achievements (Carson et al., 2003). Likewise, individuals with a broader attentional focus, who tend to suffer from distraction by irrelevant or peripheral stimuli, have been found to produce more and better creative output (e.g., poetry; Kasof, 1997). These findings, though indirect, suggest that a tendency toward mind wandering is beneficial for creativity.

Direct evidence that mind wandering can have a beneficial effect on creativity comes from a study that investigated the effects of dispositional as well as situational mind wandering in a creative incubation paradigm (Baird et al., 2012). To assess creativity, participants were asked to generate original ideas for unusual uses of objects (alternate uses task). In-between blocks of this creativity task, participants were interrupted for 12 min (serving as an incubation interval) and asked to perform a different task. This task varied in how demanding it was, and thus how conducive to mind wandering. The results showed that the greatest increase in creativity post-incubation occurred when participants had performed an undemanding task, which left room for mind wandering. In addition, the study showed that greater dispositional mind wandering, assessed with a questionnaire, was associated with greater creativity overall. This finding is in line with a more recent survey study showing that an increased tendency to mind wander was associated with increased self-reported creative behavior and engagement in creative activities (Baas, 2015). Though the process underlying this relationship is not entirely exposed, Baird et al. (2012) have proposed that mind wandering may support creativity by increasing unconscious associative processing, which is thought to facilitate the generation of novel ideas or untypical solutions to problems.

## Mindfulness and Creativity

In contrast to these findings, there is also evidence linking mindfulness to greater creative performance. This evidence comes from a set of studies by Ostafin and Kassman (2012), who investigated both dispositional and situational mindfulness in relation to creative problem solving. To assess creative problem-solving, the authors used verbal puzzles (so-called insight problems) that are initially hard to solve due to misleading problem representations and require restructuring, or viewing an element of the puzzle in a different way (e.g., Ohlsson, 1992; Schooler and Melcher, 1995). In a first study, Ostafin and Kassman (2012) showed that a greater tendency toward mindfulness, assessed through the Mindful Attention Awareness Scale (MAAS; Brown and Ryan, 2003), was associated with an increased chance of solving the puzzles. In a second study, the authors showed that a brief session of mindfulness meditation improved both situational mindfulness and problem solving. The authors concluded that mindfulness benefits creativity, possibly because mindful attention to present moment experience reduces the tendency toward habitual responses when searching for the solution to a creative problem.

In a more recent study investigating the relationship between mindfulness and creativity, Baas et al. (2014) distinguished between different aspects of mindfulness. They found that the ability to focus attention and act with full awareness (assessed by the MAAS) was associated with decreased performance on the alternate uses task. However, another aspect of mindfulness—the ability to observe and attend to various stimuli—was associated with increased creativity. A study by Colzato et al. (2012) found similar results, this time focusing on mindfulness practice rather than differences in trait mindfulness. They found that mindfulness practice that involved open monitoring meditation (which promotes the ability to observe and attend to various stimuli) increased creativity in an idea generation task, whereas focused-attention meditation (training the ability to focus attention and awareness) had no effects on creativity.

## Distinct Routes to Achieving Creative Solutions

To make sense of these mixed results, we think that it is important to define creativity not only in terms of the quality of creative output (e.g., an original idea; an untypical solution to a problem), but in terms of the processes through which this output is achieved. Research examining creativity in terms of creative processes has shown that people can achieve creativity through very distinct routes (e.g., Kounios and Beeman, 2009; Nijstad et al., 2010). This point can be illustrated by the following testimonies from creative individuals asked about their creative process. First, this is how Suzanne Collins, novelist and author of the bestselling book series *The Hunger Games*, describes the process that led to her idea for the books’ story of youth fighting death matches:

“I was lying in bed, and I was channel surfing between reality TV programs and actual war coverage. On one channel, there’s a group of young people competing for I don’t even know; and on the next, there’s a group of young people fighting in an actual war. I was really tired, and the lines between these stories started to blur in a very unsettling way. That’s the moment when Katniss’s story came to me.” (Margolis and Collins, 2008)

In contrast, this is how author Erik Larson describes how he got the idea for his bestselling “non-fiction novel” *Devil in the White City*, which tells the story of the historical serial murderer H. H. Holmes:

“I was in the search then for a book idea, and I thought, well, wouldn’t it be interesting to try to do a real historical murder, do a non-fiction book about a historical murder and try and evoke some of the same effects, some of the same sort of sense of the past? And so I actually just quite systematically began looking for a good murder to write about. You don’t get more systematic than looking at—my first book from the library was the ‘Encyclopedia of Murder.’ And so seven letters in, I came to Holmes.” (Lamb and Larson, 2003)

Collins’ testimony illustrates the process of having a creative *insight*, often also referred to as an Aha-experience. Insight is characterized by the sudden realization of a novel idea or solution (“the *moment* when Katniss’s story *came to me*”; emphasis added). While to the individual an insight can seem to come out of nowhere, research suggests that it is the product of unconscious mental activity, involving the spreading of activation in semantic memory and the re-combination of information (Mednick, 1962; Jung-Beeman et al., 2004; Kounios et al., 2008), which gives rise to awareness only once an idea or solution is found (Schooler et al., 1994; Schooler and Melcher, 1995; Smith and Kounios, 1996; Bowden and Jung-Beeman, 2003). In stark contrast to this process is that described in Larson’s testimony, which is a more *analytic strategy*. It involves the deliberate search for an idea or solution, during which the individual is aware not just of the output of the search but also of its incremental progress, involving, for instance, conscious reasoning and considering or rejecting inadequate ideas or wrong solutions (Ericsson and Simon, 1993; Kounios et al., 2008).

Previously, insight and analytic problem solving have often been studied in isolation, or by comparing performance on different tasks. For instance, researchers have sometimes used insight problems as a designated tool to investigate creative insights and logic problems to investigate analytic strategic problem solving (e.g., Ansburg and Hill, 2003; see also Ostafin and Kassman, 2012). Findings that emerged from this methodological approach have supported the idea that insight and analytic problem solving involve qualitatively different processes. For instance, only insight, but not analytic problem solving suffered from verbalizing one’s thought processes (Schooler et al., 1993; Schooler and Melcher, 1995). However, while insight problems may lend themselves more to insight solving than logic problems, there is evidence that some insight problems can be solved through analytic strategy as well (e.g., Weisberg, 1986; MacGregor et al., 2001; Bowden et al., 2005). Thus, an alternative way to compare insight and analytic problem solving is to use one type of task that can be solved equally well through insight as through analytic strategy.

A frequently used task that fits this requirement (and that has shown to correlate with performance on insight problems; Schooler and Melcher, 1995), is solving compound remote associates problems (CRA problems; e.g., Mednick, 1962; Bowers et al., 1990; Kounios and Beeman, 2009). CRA problems consist of word triads (e.g., “board, magic, death”), of which each word can all be combined with a single fourth word (“black”) to form a compound word or phrase (“black board,” “black magic,” “black death”). Participants are presented with the three target words together and asked to generate the common fourth word in a limited amount of time. What makes these problems difficult to solve is that the solution is only weakly associated with each of the target words, and retrieving it from memory requires accessing a broad semantic network of associations, while inhibiting habitual responses, or strongly associated words that are not the solution (Fiore et al., 2001; Harkins, 2006; Benedek and Aljoscha, 2013).

A common observation with regard to CRA problems is that participants sometimes report having found a solution through insight, by simply letting the answer “come to them,” and sometimes through an analytic search strategy. Moreover, these self-reports are typically associated with different kinds of errors. When people approach problems in an insightful manner, they tend to make frequent errors of omission (timing out or failing to provide any answer), but relatively infrequent commission errors (providing an incorrect response). In contrast, when problems are approached with analytic strategy, they tend to make frequent commission errors but infrequent omission errors (Kounios et al., 2008). This pattern is in line with the idea that while analytic solutions are achieved gradually, with conscious awareness of potential (but often wrong) solutions under consideration, insight solutions come to conscious awareness suddenly, in an all-or-nothing manner, without access to intermediate steps or wrong solutions.

## Neuro-Cognitive Processes in Insight and Analytic Problem Solving

Evidence from behavioral studies suggests that unconscious spreading of semantic associations takes place rapidly and spontaneously upon being presented with CRA problems. For instance, when shown word triads that either comprised solvable CRA problems or were simply incoherent, “insolvable” triads, within few (2–12) seconds, participants were able to make accurate intuitive judgments about whether a triad was a solvable CRA problem or not without being able to report the solutions to the solvable problems (Bowers et al., 1990; Baumann and Kuhl, 2002; Bolte et al., 2003; Bolte and Goschke, 2005; Topolinski and Strack, 2008, 2009). Other studies have demonstrated that presenting CRA problems “primed” their solution, as evidenced by faster recognition of the solution word (e.g., Beeman et al., 1994). Interestingly, research has found that this semantic spreading process was disrupted when individuals followed instructions to actively search for the problem solutions, which likely induced a more analytic approach to the problems (Topolinski and Strack, 2008). These findings are in line with the notion that qualitatively different cognitive mechanisms (i.e., unconscious spreading of information vs. conscious search) are involved in insight and analytic problem solving.

There is also evidence from functional magnetic resonance imaging (fMRI) and electroencephalography (EEG) studies showing that participants’ self-reports of whether they approached a CRA problem with an insight approach or analytic strategy correspond to differences in neural activity that can be detected already before a solution is found, and even before a problem is initially presented to the participant (Bowden and Jung-Beeman, 2003; Kounios et al., 2006; Kounios and Beeman, 2009). The fact that the activity precedes the process of solving the problems suggests that it corresponds to different ways of approaching a problem, not to responses to its solution. Specifically, it has been suggested that activity leading up to insight solutions (which is most prominent in the temporal lobes and mid-frontal cortex, and specifically in the anterior cingulate cortex) is involved in priming the brain to process lexical and semantic information, and to detect weakly activated, subconscious solutions, and switch attention to them when they are detected (Aziz-Zadeh et al., 2009; Kounios and Beeman, 2009). In contrast, activity leading up to solutions found through analytic strategy (which is most prominent in the posterior, or visual cortex) has been suggested to correspond to focusing attention outward and toward the presented task stimuli (Kounios and Beeman, 2009).

More recently, a behavioral study has demonstrated that the way people direct their attention before attempting to solve CRA problems affects their likelihood to solve them through insight or analytic strategy (Wegbreit et al., 2014). Participants were first asked to perform one of two visual attention tasks, which differed in how broad or narrow of a focus of attention they required (i.e., an object identification task, which required participants to pay attention to a broad space, or a flanker task, which required them to focus their attention narrowly and ignore stimuli in the periphery). If participants had focused their attention broadly, they solved more subsequent problems with insight than when they had focused their attention narrowly.

Other research has focused on more stable, or trait-related individual differences. A study by Kounios et al. (2008) investigated resting-state brain activity, recorded through EEG during a period of rest, and found that different patterns of activity corresponded to tendencies to successfully solve CRA problems through insight or analytic strategy (Kounios et al., 2008). Participants who more frequently solved problems through insight (rather than analytically) showed greater right-hemisphere activity and more diffuse activity (i.e., increased in the center and decreased in surrounding areas) in the occipital cortex. This kind of diffuse activation was speculated to correspond with creative individuals’ tendency of to have a broad attentional focus and suffer from greater distractibility (Kasof, 1997; Ansburg and Hill, 2003; Carson et al., 2003; Fink et al., 2012).

## The Present Research

Taken together, these findings suggest that solving the same types of problems through insight versus strategically is associated with different neuro-cognitive processes and different attentional styles, solving through insight being more strongly associated with diffuse or broad attention and solving through analytic strategy being more strongly associated with outwardly focused attention. Based on this research, we predicted that mindfulness and it’s opposing construct mind wandering relate differently to creative problem solving as a function of whether a problem is approached through insight or analytic strategy. Specifically, we predicted that individuals with a greater tendency toward mindfulness (or a lesser tendency to mind wander) should show increased performance when approaching problems with analytic strategy, but decreased creative problem solving performance when approaching problems through insight (henceforth summarized as “differential relationship prediction”).

Two studies were conducted to test this prediction. Our investigation was based on the premise that mind wandering and mindfulness can be conceptualized as the polar ends of a continuum (Mrazek et al., 2012b). In line with this premise, individual differences in mindfulness/mind wandering were assessed using a self-report scale of mindful awareness and attention (MAAS). Creative problem solving was assessed using CRA problems. In addition to solution accuracy, we also assessed the types of errors participants made. In Study 1, problem-solving styles were measured by asking participants at the end of each trial to what degree they had approached the current problem through analytic strategy or insight (see Kounios and Beeman, 2009). In Study 2, we went a step further and explicitly instructed participants to employ a particular problem-solving approach when attempting to solve CRA problems. Both studies have were approved by the university’s internal review board were conducted in accordance with the Declaration of Helsinki.

## Study 1

### Method

#### Participants

Participants were recruited using Amazon’s Mechanical Turk. Seventy-six participants completed the study. The only exclusion criterion was that participants were excluded from analysis if they failed to solve a single remote associates problem. Six participants were removed for that reason. Of the 70 participants included in the analysis, 15 failed to complete the demographic questions. Demographic data from those who answered the questions (16 male, 39 female) indicated an average age of 36 years (SD = 13; minimum = 19, maximum = 66). All participants were residing in the USA. Education levels reached from high school to Ph.D. degrees. The mean education level was equivalent to an associate’s or bachelor degree.

#### Procedure and Materials

First, before engaging in solving a set of CRA problems, participants received the following instructions to communicate the difference between insight and analytic strategy as distinct problem-solving approaches: “There are different ways of solving these puzzles. Sometimes, they are solved with STRATEGY. That is, you try out a word without knowing whether it is the right answer, but after thinking about it strategically (e.g., trying to combine the word with each of the three given words), you figure out that it is indeed the right answer. Other times, problems are solved with what we call INSIGHT. That is, a word may come to mind spontaneously, it just pops into your head, and you immediately recognize that it is the right answer. We are interested in the way people come to solutions in this task. Therefore, after entering an answer you will be asked to indicate to what degree you found the answer through strategy or insight.”

Participants received 30 CRA problems (see **Table 1** for a list of the problems), which were presented in a random order. Each trial started with the presentation of three target words, and the instruction “you have 1 min to solve the problem,” along with a text box in which participants could type their answer. The target words, instruction line, and text box remained on the screen for 30 s. During this time, participants were able to type in the text box and revise their typed response as much as they liked. After 30 s, the problem would disappear. Thus, within 30 s, participants could either provide the correct answer, an incorrect answer (defined as commission error), or time out (i.e., provide no answer, defined as omission error). After this time window, participants were presented with an insight rating scale (adapted from Bowden and Jung-Beeman, 2003) to indicate the problem-solving style with which they had approached the problem. Because a CRA problem may not necessarily be approached exclusively with insight or analytic strategy, but potentially also with a combination of the two, we gave participants four answer options: 1 = just strategy; 2 = mostly strategy; 3 = mostly insight; 4 = just insight (see Bowden and Jung-Beeman, 2003; Chein and Weisberg, 2014).

**Table 1:** Compound remote associate (CRA) problems used in Study 1.

Word 1	Word 2	Word 3	Solution	Mean accuracy (SD)	Mean insight rating (SD)
Ache	Hunter	Cabbage	Head	0.51 (0.50)	2.64 (1.20)
Barrel	Root	Belly	Beer	0.76 (0.43)	3.29 (1.02)
Bass	Complex	Sleep	Deep	0.20 (0.40)	2.13 (1.09)
Big	Leaf	Shade	Tree	0.71 (0.46)	2.71 (1.12)
Bite	Monkey	Widow	Spider	0.53 (0.50)	2.61 (1.28)
Blade	Witted	Weary	Dull	0.23 (0.42)	2.17 (1.13)
Board	Magic	Death	Black	0.31 (0.47)	2.53 (1.13)
Broken	Clear	Eye	Glass	0.54 (0.50)	2.59 (1.17)
Chocolate	Fortune	Tin	Cookie	0.76 (0.43)	3.01 (1.10)
Falling	Actor	Dust	Star	0.57 (0.50)	2.61 (1.20)
Foot	Collection	Out	Stamp	0.09 (0.28)	2.09 (1.05)
Gold	Stool	Tender	Bar	0.46 (0.50)	2.69 (1.23)
Hall	Car	Swimming	Pool	0.67 (0.47)	3.14 (1.03)
Jump	Kill	Bliss	Joy	0.16 (0.37)	2.10 (1.18)
Measure	Desk	Scotch	Tape	0.73 (0.45)	2.64 (1.19)
Mouse	Sharp	Blue	Cheese	0.51 (0.50)	2.37 (1.29)
Off	Trumpet	Atomic	Blast	0.10 (0.30)	2.09 (1.11)
Playing	Credit	Report	Card	0.79 (0.41)	3.19 (1.08)
Pure	Blue	Fall	Water	0.41 (0.50)	2.57 (1.17)
Rabbit	Cloud	House	White	0.33 (0.47)	2.23 (1.20)
Rock	Times	Steel	Hard	0.39 (0.49)	2.34 (1.19)
Room	Blood	Salts	Bath	0.59 (0.50)	2.59 (1.16)
Salt	Deep	Foam	Sea	0.54 (0.50)	3.10 (1.00)
Sandwich	Golf	Foot	Club	0.60 (0.49)	2.70 (1.12)
Square	Cardboard	Open	Box	0.74 (0.44)	2.64 (1.10)
Stick	Light	Birthday	Candle	0.67 (0.47)	2.79 (1.20)
Strap	Pocket	Time	Watch	0.70 (0.46)	2.70 (1.13)
Surprise	Wrap	Care	Gift	0.24 (0.43)	2.34 (1.10)
Thread	Pine	Pain	Needle	0.61 (0.49)	2.80 (1.18)
Walker	Main	Sweeper	Street	0.67 (0.47)	2.71 (1.18)
Accuracy scores and insight ratings collapsed over problems				0.50 (0.21)	2.60 (0.33)

Mean accuracy and insight ratings per problem (SD reported in parentheses).

After the CRA problems, we assessed participants’ tendency toward mindfulness/ mind wandering, using the MAAS, a 15-item questionnaire (Brown and Ryan, 2003). A sample item is, “I break or spill things because of carelessness, not paying attention, or thinking of something else.” For the full scale, see Brown and Ryan (2003). Higher scores on the MAAS are indicative of greater mindfulness, or a reduced tendency to mind wander.

### Results

#### Main analyses

##### Predicting accuracy and error types from MAAS scores

Reliability of the MAAS in this sample was high (Cronbach’s alpha = 0.89). We performed regression analyses to test whether MAAS scores predicted accuracy and error types. We first tested whether MAAS scores predicted overall performance on CRA problems, regardless of problem-solving style. This was the case. Higher MAAS scores predicted lower overall accuracy, β = -0.25, *t*(69) = -2.14, *p* = 0.04. This effect appeared to be driven by increased errors of commission, β = 0.31, *t*(68) = 2.65, *p* = 0.01, whereas no relationship was found between MAAS scores and errors of omission, β = -0.15, *t*(68) = -1.22, *p* = 0.23.

**FIGURE 1:**
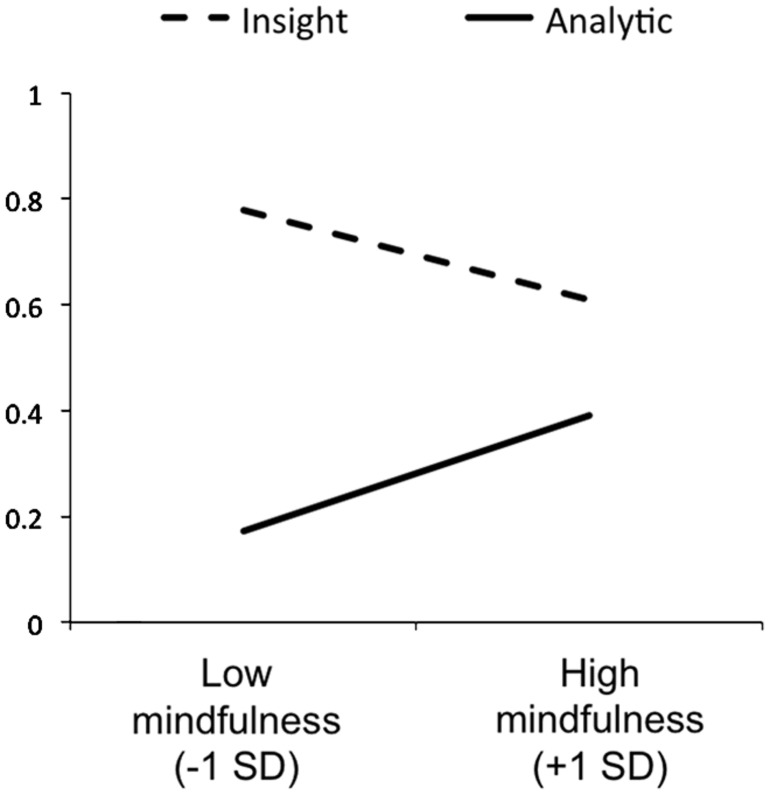
**Regression lines illustrating the relationship between mindfulness scores and accuracy for compound remote associate (CRA) problems reported to have been approached exclusively with insight and problems reported to have been approached exclusively with analytic strategy**.

Next, we tested our specific prediction of a differential relationship between MAAS scores and creative problem-solving performance as a function of problem-solving style. For problems approached exclusively with insight (problems rated 4 on the insight rating scale), higher MAAS scores predicted lower accuracy, β = -0.25, *t*(66) = -2.04, *p* = 0.045 (see **Figure 1**). Moreover, for these problems, higher MAAS scores predicted more commission errors, β = 0.31, *t*(62) = 2.64, *p* = 0.01, but were unrelated to omission errors, β = -0.01, *t*(65) = -1.01, *p* = 0.92. In contrast, for problems approached exclusively with analytic strategy (problems rated 1 on the insight rating scale), higher MAAS scores predicted higher accuracy, β = 0.36, *t*(63) = 3.03, *p* = 0.04 (see **Figure 1**). Moreover, MAAS scores were not related to commission errors, β = 0.14, *t*(63) = 1.11, *p* = 0.27, but predicted fewer omission errors, β = -0.38, *t*(63) = -3.22, *p* = 0.002. Thus, taken together, the present results show that individual differences in mind wandering/mindfulness were related to general creative performance as well as how effective (in terms of accuracy) a given problem-solving style was for participants.

For completeness, we also report the results for problems less exclusively approached with insight or analytic strategy: When participants reported having approached problems mostly with insight (score 3 on the insight rating scale), MAAS scores did not significantly predict accuracy, β = -0.17, *t*(62) = -1.35, *p* = 0.18, or commission errors, β = 0.01, *t*(62) = 0.07, *p* = 0.94, but higher MAAS scores were associated with more omission errors, β = 0.32, *t*(62) = 2.66, *p* = 0.01. When participants reported having approached problems mostly with analytic strategy (score 2 on the insight rating scale), MAAS scores did not significantly predict accuracy, β = -0.16, *t*(60) = -1.24, *p* = 0.22, nor errors of commission, β = 0.13, *t*(60) = 1.01, *p* = 0.32, or omission, β = 0.06, *t*(60) = 0.49, *p* = 0.62.

#### Additional analyses

##### Predicting problem-solving styles from MAAS scores

Since individual differences in mind wandering/mindfulness predicted the *success* (i.e., accuracy; specific errors) of problem solving as a function of problem-solving style, one might ask whether these individual differences also predicted which problem-solving style was favored by participants. Average frequencies of the four different insight ratings were as follows: 1 (“just strategy”): *M* = 7.53, SD = 5.26; 2 (“mostly strategy”): *M* = 6.00, SD = 4.38; 3 (“mostly insight”): *M* = 6.83, SD = 4.89; 4 (“just insight”): *M* = 9.64, SD = 5.72. Thus, participants used the scale ends somewhat more often than the middle categories. Overall, there was a slight trend for participants to more frequently approach problems in an insightful manner; insight ratings (*M* = 2.60, SD = 0.48) differed marginally significantly from the hypothetical scale midpoint *t*(69) = 1.82, *p* = 0.07. A regression analysis with MAAS scores as the independent and problem-solving style as the dependent variable showed that there was no relationship between MAAS scores and participants’ preferred problem-solving style, β = -0.05, *t*(69) = -0.43, *p* = 0.67.

##### Insight ratings and problem solution rates

Examination of the insight ratings and solution rates for each CRA problem (listed in **Table 1**) led to the observation that there was a positive correlation between the two (*r* = 0.85). Also, as can be seen in **Figure 1**, accuracy was noticeable higher for problems approached exclusively with insight (problems rated 4 on the insight rating scale; *M* = 0.63, SD = 0.38) than for problems solved exclusively with analytic strategy (problems rated 1 on the insight rating scale; *M* = 0.25, SD = 0.26), *F*(1,68) = 45.42, *p* < 0.001. Since the likelihood of solution success might be an indicator of problem difficulty, this correlation raises the question of whether problem difficulty might account for the differential relationship between mindfulness/mind wandering and problem solving with insight versus analysis. To investigate this possibility, we grouped problems into easy and difficult problems using a median split, and then repeated our critical analyses. We still found that higher MAAS scores still predicted lower accuracy for problems solved with insight (rated 4 on the insight scale), whether problems were relatively easy [β = -0.22, *t*(61) = -1.73, *p* = 0.09; marginally significant) or relatively difficult [β = -0.29, *t*(62) = -2.42, *p* = 0.02]. Moreover, higher MAAS scores still predicted greater accuracy for problems solved analytically (rated 1 on the insight scale), whether the problems were relatively easy [β = 0.31, *t*(57) = 2.47, *p* = 0.02], or relatively difficult [β = 0.29, *t*(60) = 2.30, *p* = 0.03]. Thus, we rule out problem difficulty as an alternative explanation for the differential relationship between mindfulness/mind wandering and insight versus analytic problem solving.

### Discussion

The results from Study 1 indicated that, overall, a greater tendency toward mindfulness (or a reduced tendency to mind wander) was associated with decreased problem-solving performance. However, closer examination of the data based on self-reported problem-solving approaches showed that this negative relationship was present only for problems approached exclusively with insight, while a positive relationship between mindfulness and problem solving performance was found for problems approached with analytic strategy. These results confirm our differential relationship prediction.

Examination of the types of errors participants made revealed a similarly divergent pattern; Higher mindfulness (or a reduced tendency to mind wander) was associated with more frequent commission errors overall. This remained the case for problems approached with insight, but not for problems approached with analytic strategy. This pattern of results can be interpreted to suggest that participants high in mindfulness did not rely as much on unconscious associative processes (which are associated with omission errors) when attempting to solve creative problems—and in particular when approaching them with insight—but may instead have relied more strongly on accessing conscious information (which leads to commission errors; Kounios et al., 2008). This tendency may explain why the insight approach is not a successful approach for people high in mindfulness.

A potential limitation of Study 1 was that problem-solving approaches were classified based on participants’ *post hoc* self-reports. While previous research shows that such self-reports are reliable in that they correspond to distinct patterns of neural activity taking place even before a report is made (Bowden and Jung-Beeman, 2003; Kounios et al., 2006; Kounios and Beeman, 2009), it remains a possibility that the self-reports in the present study were influenced by thought processes that took place during or before the problem-solving process but are not necessarily directly involved in the act of problem solving. For instance, participants may have inferred having approached a problem in an insightful manner if they experienced feelings of fluency or positive affect (see Subramaniam et al., 2009; Topolinski and Reber, 2010). Moreover, although we did not find that differences in mindfulness/mind wandering predicted differences in participants’ dominant problem solving styles, it is possible that differences related to mindfulness/mind wandering could affect the reliability of participants’ self-reports. Therefore, In Study 2, we chose to experimentally manipulate problem-solving styles by instructing participants to approach problems in an insightful or analytic manner.

## Study 2

Most previous studies have only measured rather than instructed problem-solving styles in CRA problems. However, in one previous study, participants were presented with both solvable CRA problems and incoherent word triads and were either instructed to search for a solution word, or to simply read or read and memorize the word triads (Topolinski and Strack, 2008). Search instructions eliminated spontaneous semantic spreading (as evidenced by reduced priming of solution words). This can be taken to suggest that instructions to search for the solution reduces the likelihood of spontaneous insights. In other studies, more specific explicit instructions have been used to induce different performance strategies on tasks that did not—as with CRA problems—involve the internal search for a solution in memory, but the search of an external stimulus among other stimuli. Smilek et al. (2006b), for example, (see also Snodgrass et al., 1995; Smilek et al., 2006a) aimed to manipulate participants’ cognitive strategies when searching for a visual target among distractors. Participants were either instructed to remain passive and simply let the target item “pop into their mind”—a process strongly reminiscent of an insight experience—or to “direct their attention” in a more controlled way and “search” actively for the target—a process resembling the analytic approach to creative problem-solving. The researchers found that participants performed better when they used the passive, or insight, approach then when actively searching for the target.

We do not imply the same cognitive processes involved in the internal search for a solution in memory are involved in searching for stimuli in the external environment. However, these studies compellingly demonstrate that explicit instructions can influence how people approach search tasks. We used instructions similar to those by Smilek et al. (2006b) to induce an insightful versus analytic approach to solving CRA problems. As in Study 1, we predicted that individuals with a greater tendency toward mindfulness would perform better when approaching problems with analytic strategy, but worse when approaching problems through insight. To test whether one or both types of instructions led to changes in task performance, we also included a control condition in which participants received no instructions regarding their problem-solving approach.

### Method

#### Participants

Participants were again recruited using Amazon’s Mechanical Turk. Hundred-twenty participants completed the study. As in Study 1, participants were excluded from analyses if they failed to solve a single remote associates problem. Twenty-one participants were removed for that reason, leaving 99 participants in the analyses (42 male, 57 female, average age = 38 years, SD = 12). All participants resided in the USA. The average education level was equivalent to a bachelor’s degree.

#### Design

The study employed the following nested design. (1) Participants were assigned to one of two groups; a control group, which did not receive instructions regarding their problem-solving approach, and an instruction group, which did receive instructions. The choice for this between-group manipulation was made in order to have a “naïve” control group, which is not influenced by knowledge of or experience with different problem-solving instructions. (2) Instructions to employ an insight or analytic problem-solving approach were varied within participants in the instruction group; Participants received one type of instruction for a first block of the problem-solving task, and the other type of instruction for the second block. The order in which the two types of instructions were presented was counterbalanced. This within-participants manipulation served to reduce confounds between the instructed problem-solving style and pre-existing individual differences in problem-solving styles or ability.

#### Procedure and Materials

Creative problem solving was again measured using the CRA task. After general introductory task instructions, participants in the *control group* directly continued to the task. In the *instruction group*, participants received the following additional instructions before continuing to the task: “There are different approaches to solving this task. In the current study, we want to find out which one is the most effective. We divided the task into two blocks. We would like you to use a different approach in each block. Each approach will be explained in detail before the beginning of the block. Please try to follow the instructions as closely as possible, even if you find that the instructed strategy does not feel natural or effective to you.”

Before each block of CRA problems, participants in the instruction condition received one of the following instructions (underlined parts highlight words specific to the insight vs. analytic approach). Insight approach:

“One way to solve this task is to be as receptive and as possible and let the right answer “pop” into your mind. The idea is to let your intuition determine your response. For instance: Do not try to make exhaustive mental lists of words associated to each of the three words on the screen. Instead, just look at the words on the screen and wait for potential answers to “pop up.” Sometimes people find it difficult or strange to tune into their “gut feelings,” but we would like you to try your best. Remember, it is very critical for this experiment that you respond intuitively and let the answer just “pop” into your mind.”

Analytic strategy approach:

“One way to solve this task is to be as active and as possible and to “search” for the right answer. The idea is to let systematic effort and strategy determine your response. For instance: Try to make exhaustive mental lists of words associated to each of the three words on the screen. Start with the first word on the screen. List all the associated words you can think of. Then move to the second and then the third word on the screen and do the same. Sometimes people find it difficult or strange to “search systematically,” but we would like you to try your best. Remember, it is very critical for this experiment that you actively and systematically search for the answer.”

All participants were presented with 48 CRA problems split into two blocks of 24 (see **Table 2** for the stimulus materials). The problems were presented in randomized order, and the presentation format of the problems was the same as in Study 1. At the end of the task, participants in the instruction condition were asked to report on a 7-point scale how natural they found each problem-solving approach and how effective they thought each approach was. Finally, we again administered the MAAS (Brown and Ryan, 2003).

**Table 2:** Compound remote associate problems used in Study 2.

Word 1	Word 2	Word 3	Solution	Mean accuracy (SD)
Ache	Hunter	Cabbage	Head	0.60 (0.49)
Bass	Complex	Sleep	Deep	0.23 (0.42)
Big	Leaf	Shade	Tree	0.74 (0.44)
Blade	Witted	Weary	Dull	0.23 (0.42)
Board	Magic	Death	Black	0.40 (0.49)
Broken	Clear	Eye	Glass	0.59 90.50)
Chamber	Mask	Natural	Gas	0.47 (0.50)
Chocolate	Fortune	Tin	Cookie	0.80 (0.40)
Date	Alley	Fold	Blind	0.32 (0.47)
Dive	Light	Rocket	Sky	0.23 (0.42)
Dream	Break	Light	Day	0.53 (0.50)
Envy	Golf	Beans	Green	0.64 (0.48)
Falling	Actor	Dust	Star	0.73 (0.45)
Folk	Bird	Swan	Song	0.63 (0.49)
Force	Line	Mail	Air	0.20 (0.40)
Foul	Ground	Mate	Play	0.17 (0.38)
Fox	Man	Peep	Hole	0.41 (0.50)
Hall	Car	Swimming	Pool	0.74 (0.44)
Health	Taker	Less	Care	0.31 (0.47)
Hound	Pressure	Shot	Blood	0.49 (0.50)
Lift	Light	Rocket	Sky	0.42 (0.50)
Magic	Plush	Floor	Carpet	0.68 (0.47)
Manners	Round	Tennis	Table	0.71 (0.46)
Measure	Desk	Scotch	Tape	0.81 (0.40)
Mouse	Sharp	Blue	Cheese	0.55 (0.50)
Nuclear	Feud	Album	Family	0.54 (0.50)
Peach	Arm	Tar	Pit	0.44 (0.50)
Playing	Credit	Report	Card	0.76 (0.43)
Rabbit	Cloud	House	White	0.43 (0.50)
Right	Cat	Carbon	Copy	0.31 (0.47)
River	Note	Account	Bank	0.62 (0.49)
Rock	Times	Steel	Hard	0.68 (0.47)
Salt	Deep	Foam	Sea	0.58 (0.50)
Sandwich	Golf	Foot	Club	0.48 (0.50)
Sea	Home	Stomach	Sick	0.51 (0.50)
Shopping	Hand	Wheel	Cart	0.71 (0.46)
Sleeping	Bean	Trash	Bag	0.76 (0.43)
Sore	Shoulder	Sweat	Cold	0.23 (0.42)
Square	Cardboard	Open	Box	0.80 (0.40)
Strap	Pocket	Time	Watch	0.77 (0.42)
Surprise	Wrap	Care	Gift	0.25 (0.44)
Test	Runner	Map	Road	0.33 (0.47)
Thread	Pine	Pain	Needle	0.69 (0.47)
Trip	House	Goal	Field	0.19 (0.40)
Type	Ghost	Screen	Writer	0.48 (0.50)
Way	Board	Sleep	Walk	0.30 (0.46)
Widow	Bite	Monkey	Spider	0.68 (0.47)
Zone	Still	Noise	Quiet	0.33 (0.47)
Insight scores collapsed over problems				0.51 (0.15)

Mean accuracy per problem (SD reported in parentheses).

### Results

#### Main analyses

##### Predicting accuracy and error types from MAAS scores

**FIGURE 2:**
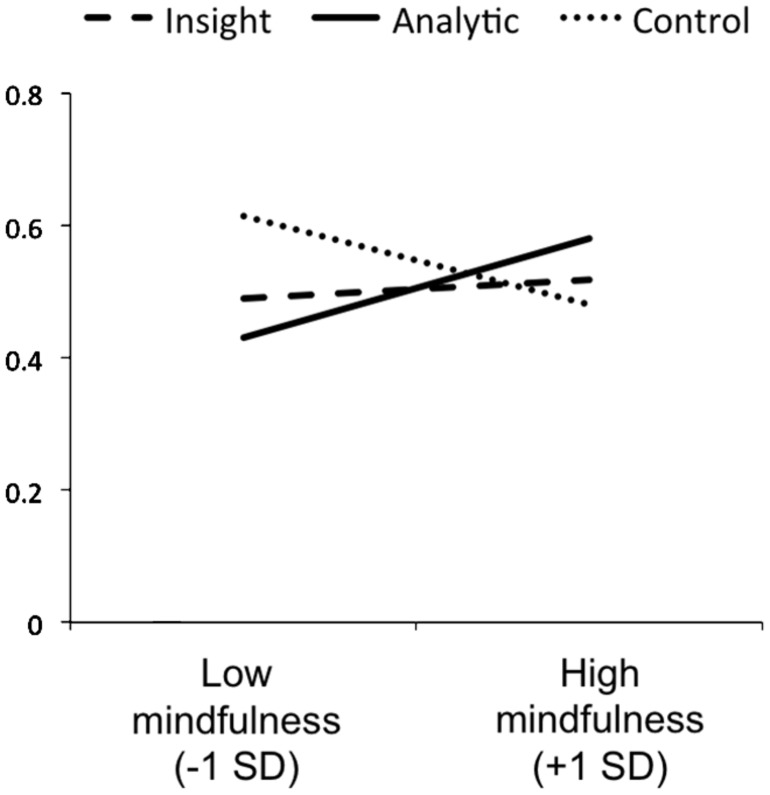
**Regression lines illustrating the relationship between mindfulness scores and accuracy for CRA problems in the control condition, following insight instructions, and following analytic instructions**.

Reliability of the MAAS in this sample was high (Cronbach’s alpha = 0.90). We performed regression analyses to test whether MAAS scores predicted accuracy and error types in the different conditions. First, in the control group, we found that greater MAAS scores predicted lower accuracy, β = -0.43, *t*(32) = -2.63, *p* = 0.01 (see **Figure 2**). MAAS scored did not significantly predict errors of commission, β = 0.28, *t*(31) = 1.61, *p* = 0.12, or omission, β = 0.08, *t*(31) = 0.44, *p* = 0.66.

Next, we tested our specific hypothesis that individual differences in the tendency to mind wander or be mindful differently predict creative performance depending on the problem-solving style used to approach a problem. Following analytic strategy instructions, higher MAAS scores predicted greater accuracy β = 0.27, *t*(62) = 2.21, *p* = 0.03 (see **Figure 2**). Moreover, higher MAAS scores predicted reduced commission errors, β = -0.29, *t*(64) = -1.39, *p* = 0.02, and were unrelated to omission errors, β = 0.10, *t*(64) = 0.82, *p* = 0.42. Following insight instructions, MAAS scores were unrelated to accuracy, β = 0.09, *t*(62) = 0.72, *p* = 0.47 (see **Figure 2**). Moreover, higher MAAS scores predicted reduced commission errors, β = -0.26, *t*(64) = -2.18, *p* = 0.03, but increased errors of omission, β = 0.30, *t*(64) = 2.47, *p* = 0.02.

#### Additional analyses

##### Effects of instructions on performance

Because using explicit instructions to induce distinct problem-solving approaches is a novel methodological approach in the context of creative problem solving, we examined how participants in the instruction group evaluated the two problem-solving approaches and whether instructions affected accuracy on the attempted CRA problems. Repeated measures ANOVAs on participants evaluations of the two approaches showed that they found the insight approach (*M* = 5.55, SD = 1.43) more *natural* than the analytic strategy approach (*M* = 3.95, SD = 1.62), *F*(1,65) = 26.62, *p* < 0.001, and that they thought that the insight approach (*M* = 5.05, SD = 1.46) was more *effective* than the analytic approach (*M* = 4.35, SD = 1.44), *F*(1,65) = 6.77, *p* = 0.01.

Next, we performed within- and between-participants analyses to test whether out instructions affected accuracy. First, a repeated-measures ANOVA with the factor instruction performed on the data from the instruction group yielded no difference in accuracy between analytic (*M* = 0.50, SD = 0.17) and insight trials (*M* = 0.50, SD = 0.16), *F*(1,65) = 0.02, *p* = 0.90. This remained the case after entering participants responses of how natural and effective they found each approach as covariates, *F*(1,65) = 0.07, *p* = 0.80. Additional repeated-measures ANOVA were performed to examine the types of errors made in the different conditions. There were not differences in commission errors between analytic (*M* = 0.34, SD = 0.03) and insight trials (*M* = 0.32, SD = 0.03), *F*(1,65) = 1.44, *p* = 0.24. However, analytic trials led to slightly more omission errors (*M* = 0.18, SD = 0.02) than insight trials (*M* = 0.16, SD = 0.02), *F*(1,65) = 4.09, *p* = 0.047.

There was no effect of the order in which the different instructions were administered on accuracy, *F*(1,64) = 0.08, *p* = 0.78, errors of commission, *F*(1,64) = 1.31, *p* = 0.26, or omission, *F*(1,64) = 3.79, *p* = 0.06, and no interaction of instruction and order on accuracy, *F*(1,64) = 0.01, *p* = 0.91, or errors of commission, *F*(1,64) = 0.55, *p* = 0.42, or omission, *F*(1,64) = 2.05, *p* = 0.16.

Moreover, an ANOVA comparing the two participants groups showed that accuracy in the instruction group (*M* = 0.50, SD = 0.14) did not differ from that in the control group who received no instructions regarding their problem-solving approach (*M* = 0.53, SD = 0.15), *F*(1,97) = 0.75, *p* = 0.39. There were also no differences in commission errors (instruction group: *M* = 0.33, SD = 0.20; control group: *M* = 0.30, SD = 0.19), *F*(1,97) = 0.57, *p* = 0.45, or omission errors (instruction group: *M* = 0.17, SD = 0.14; control group: *M* = 0.17, SD = 0.17), *F*(1,97) = 0.02, *p* = 0.89. Thus, taken together, these tests provide evidence that, while participants preferred the insight approach in their evaluations, instructions to follow any of the two approaches did not affect participants’ success at solving CRA problems or the types of errors they made.

### Discussion

Study 2 investigated the relationship between mindfulness/mind wandering and creative problem solving after different problem-solving styles were instructed rather than adopted spontaneously. When participants did not receive instructions, a greater tendency toward mindfulness (or a reduced tendency to mind wander) predicted decreased creative performance. This is consistent the results from Study 1, where we found the same relationship when analyzing performance collapsed over the different self-reported problem-solving styles. Mindfulness did not significantly predict errors observed in the no-instruction condition, though numerically the results for the types of errors made are similar to those in Study 1 collapsed over problem-solving styles, and the non-significant relationship between mindfulness and commission errors in this study may be due to a lack of power in the no-instruction condition due to the smaller sample size.

When participants received instructions to adopt a particular problem solving approach, we found that mindfulness/mind wandering was differentially related to problem solving as a function of the specific approach. When participants were instructed to approach problems with analytic strategy, a greater tendency toward mindfulness predicted enhanced performance. This finding is in line with the results of Study 1. For problems approached with insight, we found no relationship between mindfulness/mind wandering and performance. This latter finding differed from the result found in Study 1 for self-reported insight problems. However, taken together, the results are in line with the prediction that individual differences in mindfulness/mind wandering differentially predict problem-solving performance as a function of the problem solving approach.

The results for the types of errors participants made in the instruction condition also differed from those observed in Study 1. Here, we found that higher mindfulness was associated with reduced, rather than increased, commission errors, and this was true under analytic as well as insight instructions. These discrepancies in the findings of Studies 1 and 2 suggest the approaches participants adopted following our instructions were likely not identical to those adopted spontaneously (i.e., in Study 1). It is likely that instructions to approach a problem with insight may be particularly difficult to follow. Instructions to intentionally *use* a particular strategy (e.g., making mental lists of associated words) appear more concrete and hence easier to follow than instructions to *refrain from* using such a strategy (i.e., being receptive and waiting for a solution to come to mind). Difficulty following insight instructions may also explain the absence of a relationship between mindfulness and performance in the insight condition in this study. Nonetheless, the fact that our data showed that the two types of instructions led to different relationships between mindfulness and performance (as predicted), without changing average performance scores, gives some confidence that such instructions can be a useful tool to investigate the mechanisms and consequences of different problem-solving approaches.

## General Discussion

In past research, the question of how individual differences related to attention affect creative performance has received mixed answers. Some studies have provided indirect evidence (Kasof, 1997; Carson et al., 2003; White and Shah, 2006, 2011; Fink et al., 2012) and others more direct evidence (Baird et al., 2012; Baas, 2015) that a tendency toward mind wandering increases creativity. At the same time, other research suggests that mindfulness can be associated with greater creative performance (Ostafin and Kassman, 2012). Given that mind wandering and mindfulness have been characterized as opposing constructs (Mrazek et al., 2012b), the respective conclusions that each can enhance creativity would seem to be at odds with one another. However, when examining the literature closely, it appears that different mechanisms have been brought up to account for the mixed results. It has been speculated that mind wandering facilitates creativity by stimulating unconscious associative processes that can lead to a sudden insight (Baird et al., 2012). On the other hand, it has been speculated that mindfulness increases creativity because it reduces the tendency to rely on habitual responses during the search for a creative solution (Ostafin and Kassman, 2012). This latter explanation seems to imply that the analytic route is used to find a solution. Thus, both these theories may be consistent with each other when considering insight and analytic strategies as distinct paths to creative problem solving.

Based on this premise, we predicted that a reduced tendency toward mindfulness, or a greater tendency toward mind wandering, increases problem solving when problems were approached in an insightful way, but impair problem-solving when problems are approached in an analytic way. The results from the present studies support this hypothesis. While higher mindfulness scores predicted decreased creative problem solving overall (Studies 1 and 2), a more nuanced picture emerged when examining analytic and insight solutions separately. Greater mindfulness was consistently associated with increased problem solving performance when problems were approached with analytic strategy, whether this strategy was spontaneously adopted (Study 1) or instructed (Study 2). In contrast, the relationship between mindfulness and problem solving was negative when participants spontaneously adopted an insight approach (Study 1), and absent when they were instructed to approach problems with insight (Study 2).

In the present studies, we chose to both measure and experimentally manipulate approaches to creative problem solving to provide convergent evidence for our prediction from different research methods. While the two methods largely yielded similar results, some discrepancies emerged in the results related to insight solving. Moreover, both methods have different strengths and limitations, which may explain the discrepant findings.

In Study 1, we measured spontaneously adopted problem solving approaches using self-reports, because previous research has shown that such self-reports are reliable in that they correspond to different kinds of (preceding) neural activation (Bowden and Jung-Beeman, 2003; Kounios et al., 2006; Kounios and Beeman, 2009). However, we cannot rule out that these self-reports are influenced by incidental thought processes taking place during or before the problem-solving process rather than reflecting processes directly involved in the act of problem solving. Moreover, since we examined insight and analytic problem solving in relation to individual differences in mindfulness/mind wandering, a concern was that these individual differences might be related to differences in the reliability of participants’ self-reports, which would present a confound.

In Study 2, we experimentally manipulated problem-solving approaches through instructions to rule out such confounds. However, instructions can be hard to follow, especially when they try to emulate spontaneous cognitive phenomena such as having a sudden insight. While the different types of instructions in Study 2 led to different results, we suspect based on the pattern of results that the insight instructions were less effective at inducing an insight approach than the analytic instructions were in inducing analytic strategy. In conclusion, we think that the method of experimentally manipulating problem-solving approaches through instructions is a promising method for examining differences between approaches, but can be further improved, and may be most fruitful when combined with self-reports.

Our finding that an increased tendency to mind wander (or a lesser tendency to be mindful) was associated with increased insight solving in particular is in line with a recent study examining the processes that lead to real-life creative ideas in professional writers and elite theoretical physicists (Gable et al., unpublished manuscript). For 2 weeks, the writers and physicists were asked to write daily diary reports in which they wrote down their work-related ideas and responded to questions about the circumstances under which these ideas occurred. This included whether they had been mind wandering, and whether ideas were associated with an “aha” experience, or a feeling of insight. The results showed that a substantial proportion of the ideas occurred while mind-wandering, and of these ideas, the majority were associated with aha experiences. Thus, like the present findings, these results point to a specific advantage of mind wandering for achieving creative insights.

To date, most research on the consequences of mind wandering has focused exclusively on negative consequences, including impaired performance on a range of tasks (e.g., Mrazek et al., 2012a; Randall et al., 2014) and negative mood (Killingsworth and Gilbert, 2010). Only few studies have pointed to benefits of mind wandering (e.g., Baird et al., 2012; Franklin et al., 2013; Gable et al., unpublished manuscript; see also Mooneyham and Schooler, 2013). A similar imbalance exists with regard to mindfulness. Studies have demonstrated wide-ranging benefits of mindfulness, involving cognitive functioning and well-being (e.g., Brown and Ryan, 2003; Brown et al., 2007), but have mostly neglected the question of potential costs. The present research contributes to a more nuanced view on mind wandering and mindfulness by showing that there are specific operations for which mind wandering is beneficial and mindfulness costly. In a similar vein, a recent study investigated the relationship between mindfulness and intuition (Remmers et al., 2014). Participants were asked to make coherence judgments over solvable and unsolvable CRA problems, and intuition was defined as showing above-chance accuracy without having conscious access to problem solutions. The results showed that higher mindfulness was associated with reduced intuition. This result fits well with the present findings, and supports the idea that mindfulness specifically impairs performance aspects that rely on intuitive thinking or spontaneous insights. Future research may help further examine the particular cognitive operations that are facilitated or impaired by mindfulness, and thereby further our understanding of the concept.

### Future Directions

The findings as well as the limitations of the present studies point to interesting new directions for future research. For one, the present studies have focused specifically on insight and analytic search as two distinct creative processes that we expected to benefit differently from the ability to mindfully focus attention. To get a more complete understanding of the relationship between mindfulness/mind wandering and creativity, it would be useful to examine other creative processes. For instance, researchers have distinguished between idea generation and idea selection (Ritter et al., 2012). Coming up with as many creative ideas as possible likely benefits from broad attention, while selecting the best idea among the host of ideas one has generated likely requires much more focused attention. Thus, based on the current theory and findings, we would predict that idea generation should benefit from a tendency to mind wander, while idea selection should benefit from a tendency toward mindfulness. Similarly, researchers have distinguished between cognitive flexibility and persistence as two distinct routes to creative performance (Nijstad et al., 2010). We would predict that mind wandering may benefit creativity through increased cognitive flexibility, while mindfulness may benefit creativity by supporting greater persistence.

Another important task for future research is to examine how different operationalizations of mindfulness and mind wandering relate to creative processes. In the present research, we have focused only on dispositional mindfulness/mind wandering assessed through a self-report measure. This allows for potential confounds; the differences we found in insight and analytic problem solving could be explained by a third variable that is related to differences in mindfulness/mind wandering, such as working memory (e.g., McVay and Kane, 2009; De Dreu et al., 2012; Wiley and Jarosz, 2012; Mooneyham and Schooler, 2013). It would therefore be useful to investigate whether experimentally induced differences in mindfulness resulting, for instance, from mindfulness meditation exercises (Ostafin and Kassman, 2012), or differences in mind wandering resulting from different contextual demands (Baird et al., 2012) also lead to distinct benefits for insight and analytic problem solving.

On a related note, our investigation was based on a particular conceptualization of mindfulness. We defined mindfulness as mindful attention and awareness, and thus in opposition to mind wandering. There are admittedly other aspects to mindfulness that may not necessarily be related to mind wandering and to creativity in the same way. For instance, Baas et al. (2014) have demonstrated that, whereas the ability to focus attention and act with awareness was associated with decreased creativity, the ability to mindfully observe and attend to various stimuli was associated with increased creativity. Thus, it would be interesting to investigate whether the ability to mindfully observe and attend to stimuli relates differently to insight and analytic approaches to creative problem solving. Since the analytic strategy approach is largely conscious and involves considering and rejecting potential solutions, this approach might benefit from mindful observation, whereas the largely unconscious processes leading to insights might not.

## Conclusion

Researchers and laypeople alike have brought up the question of what makes some individuals more creative than others, and studies have provided an abundance of mixed and often indirect evidence linking individual differences in traits related to mind wandering or mindfulness to creativity. The present results illustrate that, to understand how such traits impact creative performance, it is important to understand creativity as a process that can be approached in different ways. While a tendency to be mindful appears to benefit creativity when a creative task is approached in a conscious, analytic way, it harms creative performance when the task is approached in a more insightful way. Thus, both our stereotypes of creative individuals highly focused individuals and as chaotic, scattered minds seem to have merit, but they speak to different creative processes.

## Conflict of Interest Statement

The authors declare that the research was conducted in the absence of any commercial or financial relationships that could be construed as a potential conflict of interest.

## Footnotes

1 We distinguish mind wandering from daydreaming, which we define as (likely deliberately) engaging in mind wandering in the absence of a primary task. It is conceivable that an individual may be highly mindful while focusing attention on an internal train of thought, or daydream.
